# The Association Between Smartphone App–Based Self-monitoring of Hypertension-Related Behaviors and Reductions in High Blood Pressure: Systematic Review and Meta-analysis

**DOI:** 10.2196/34767

**Published:** 2022-07-12

**Authors:** Aikaterini Kassavou, Michael Wang, Venus Mirzaei, Sonia Shpendi, Rana Hasan

**Affiliations:** 1 The Primary Care Unit Department of Public Health and Primary Care The University of Cambridge Cambridge United Kingdom

**Keywords:** self-monitoring, smartphone apps, behavior change, hypertension, blood pressure, mobile health, mHealth, mobile app, self-management, lifestyle

## Abstract

**Background:**

Self-monitoring of behavior can support lifestyle modifications; however, we do not know whether such interventions are effective in supporting positive changes in hypertension-related health behaviors and thus in reducing blood pressure in patients treated for hypertension.

**Objective:**

This systematic literature review evaluates the extent to which smartphone app–based self-monitoring of health behavior supports reductions in blood pressure and changes in hypertension-related behaviors. It also explores the behavioral components that might explain intervention effectiveness.

**Methods:**

A systematic search of 7 databases was conducted in August 2021. Article screening, study and intervention coding, and data extraction were completed independently by reviewers. The search strategy was developed using keywords from previous reviews and relevant literature. Trials involving adults, published after the year 2000, and in the English language were considered for inclusion. The random-effects meta-analysis method was used to account for the distribution of the effect across the studies.

**Results:**

We identified 4638 articles, of which 227 were included for full-text screening. A total of 15 randomized controlled trials were included in the review. In total, 7415 patients with hypertension were included in the meta-analysis. The results indicate that app-based behavioral self-monitoring interventions had a small but significant effect in reducing systolic blood pressure (SBP), on average, by 1.64 mmHg (95% CI 2.73-0.55, n=7301; odds ratio [OR] 1.60, 95% CI 0.74-3.42, n=114) and in improving changes in medication adherence behavior (standardized mean difference [SMD] 0.78, 95% CI 0.22-1.34) compared to usual care or minimal intervention. The review found the intervention had a small effect on supporting improvements in healthy diet by changing habits related to high sodium food (SMD –0.44, 95% CI –0.79 to –0.08) and a trend, although insignificant, toward supporting smoking cessation, low alcohol consumption, and better physical activity behaviors. A subgroup analysis found that behavioral self-monitoring interventions combined with tailored advice resulted in higher and significant changes in both SBP and diastolic blood pressure (DBP) in comparison to those not providing tailored advice (SBP: –2.92 mmHg, 95% CI –3.94 to –1.90, n=3102 vs –0.72 mmHg, 95% CI –1.67 to 0.23, n=4199, *χ*^2^=9.65, *P*=.002; DBP: –2.05 mmHg, 95% CI –3.10 to –1.01, n=968 vs 1.54 mmHg, 95% CI –0.53 to 3.61, n=400, *χ*^2^=9.19, *P*=.002).

**Conclusions:**

Self-monitoring of hypertension-related behaviors via smartphone apps combined with tailored advice has a modest but potentially clinically significant effect on blood pressure reduction. Future studies could use rigorous methods to explore its effects on supporting changes in both blood pressure and hypertension-related health behaviors to inform recommendations for policy making and service provision.

**Trial Registration:**

PROSPERO CRD42019136158; https://www.crd.york.ac.uk/prospero/display_record.php?RecordID=136158

## Introduction

Hypertension, or high blood pressure, affects over 1 billion adults globally and is a leading risk factor for premature morbidity and mortality [1,2]. However, only about half of adults with hypertension achieve adequate blood pressure control, increasing both health care resources and the cost required for treatment [3]. In England, hypertension is estimated to cost the National Health Service an excess of £2 billion (US $2.4 billion) per year [4]. Although various risk factors contribute to poorly controlled blood pressure, nonadherence to prescribed health behaviors, like compliance to prescribed medications [5], improvements in physical activity [6,7], low salt intake [8,9], consumption of fruits and vegetables [10], low alcohol consumption [11], and smoking cessation [12], independently account for most of these uncontrolled cases.

Modifying health-related behaviors to address the underlying risk factors of hypertension could result in clinically significant health improvements and reduce morbidity, mortality, and treatment cost. Practitioners have an important role in prescribing lifestyle modifications; however, the time they can spend providing advice about and supporting adherence to health behavior change recommendations is limited and expensive [13], and there is currently limited evidence on effective interventions to support health behavior change in patients treated for hypertension [14-16].

There is growing interest in the potential of digital innovations as an inexpensive and scalable method to deliver personalized advice to people with long-term health conditions, enabling them to improve adherence to their recommended health behavior modifications and achieve health improvements [17-19]. Mobile apps, facilitated via digital technologies such as computers, smartphones, tablets, and other mobile devices are accessible to large numbers of people and in different settings [20]. Smartphone apps appear to be promising due to their potential to complement physician efforts and engage patients in decision-making processes regarding their health care [21,22]. Users of app-based interventions can receive real-time advice about patterns of health behaviors that impact their long-term health condition [23], with the potential to eliminate barriers that rely on memory and are prone to inaccuracies and recall bias, and to better inform shared decision-making during usual care consultations.

Moreover, reporting and monitoring health behaviors using apps could act as a behavior change strategy to support the individual in self-regulating health behaviors and thus lead to sustained improvements in clinical health indicators [23,24]. Self-monitoring of behavior could underpin individual behavior change by modifying self-regulation processes, for example, by enabling patients to reflect on and change their health behaviors informed by behavioral performance [24-26]. Interventions providing advice to support patients’ self-regulatory processes might be more effective at improving long-term treatment adherence and thus might be a cost-effective solution for sustained health care.

While smartphone app–based self-monitoring of health behaviors has the potential to have a direct, positive effect on patients’ health and an indirect effect on service provision, to date there is a lack of evidence on the clinical effectiveness of app-based behavioral self-monitoring to support patients treated for hypertension.

Previous systematic reviews have evaluated the impact of app-based interventions to support changes in behavioral or clinical outcomes, suggesting some promising evidence of their potential effectiveness [18,27-30]. Furthermore, content analysis of publicly available apps suggests that such interventions are complex and often consist of one or a combination of the following components: generic education about the health condition, provision of social support, reminders and feedback about the behavior, feedback on blood pressure measurements, or provision of clinical advice about medicine adjustments. However, previous reviews have neither investigated the impact of behavioral self-monitoring via smartphone apps on both clinical and behavioral effectiveness nor disentangled the components that account for clinical effectiveness in patients treated for hypertension.

This review investigates whether app-based self-monitoring of health behavior reduces blood pressure and improves health behaviors in patients treated for hypertension. The review also explores the intervention components combined with the behavioral self-monitoring interventions and estimates whether and to what extent they explain intervention clinical effectiveness.

## Methods

### Systematic Searches, Study Eligibility and Selection, and Data Coding

This systematic literature review involved searching the electronic databases MEDLINE via Ovid, Embase via Ovid, Web of Science, PsycINFO, Scopus, CINAHL, and the Cochrane Central Register of Controlled Trials (CENTRAL) in August 2021 to identify eligible studies. References for additional trials involved searches in 1 additional database: JMIR Publications [31].

The search strategy was developed using keywords from previous reviews and relevant literature (see an example of the search strategy in Multimedia Appendix 1). The review included randomized controlled trials testing intervention effects on behavior change and clinical effectiveness in people treated for hypertension. Studies on trials involving adults, published after the year 2000, and in the English language were considered for inclusion. The review was preregistered on PROSPERO (CRD42019136158).

Screening of the title, abstract, and full text was conducted independently by 4 reviewers (MW, VM, SS, and RH), and disagreements were discussed by another reviewer (AK). Articles had to meet all of the following criteria to be eligible for full-text screening: (1) the population comprised adult individuals treated for hypertension; (2) the intervention consisted of self-monitoring of hypertension-related health behaviors via a mobile app; (3) the intervention aimed to support changes in both blood pressure and related health behaviors; (4) the comparator was usual care, enhanced usual care, or a minimal behavioral intervention; (5) the study included measurements of both blood pressure and health behaviors; and (6) the study design was a randomized controlled trial.

Outcome data were extracted for measurements of systolic and diastolic blood pressure (SBP and DSP, respectively), as well as health behaviors for medication adherence, physical activity, healthy diet, alcohol consumption, and smoking cessation. Outcome data for blood pressure and health behaviors were extracted for baseline and follow-up values for most of the studies; otherwise, only the follow-up values were extracted. When follow-up values were missing (eg, SD), the baseline values were selected to estimate intervention effects.

The Taxonomy of Behavior Change Techniques [32] was selected to conceptualize and guide the coding for the self-monitoring interventions. We also coded the intervention component “tailoring” for those interventions that delivered different messages to different participants based on information obtained about them [17,18], as well as the hypothesized mechanism of behavior change when these were reported. Authors of primary studies were contacted by email for missing information. Risk of bias was assessed using the Cochrane Risk of Bias tool, version 2, evaluating the risk introduced in the primary outcome of blood pressure [33,34]. Two reviewers independently coded study design and intervention components and extracted outcome data. Disagreement was discussed and resolved by a third reviewer.

### Analysis

A random-effects meta-analysis was conducted to estimate the weighted, pooled effect for each of the blood pressure and behavioral outcomes to account for the true effect that may vary across the individual studies [35]. Effect sizes for continuous outcomes were calculated using the mean difference for blood pressure and the standardized mean difference (SMD) for behavioral outcome measurements. Mean difference was selected for blood pressure because measurements used similar units, whereas SMD was selected for behavioral outcomes because measurements were obtained using diverse methods, scales, units, or a composite of these. For example, for physical activity, these were minutes of physical activity per day and number of exercise sessions per week; for medication adherence, these were days of adherence per week, summary score responses to a 5-point scale, 8-item questionnaire. Effect sizes for dichotomous outcomes were calculated using the odds ratio (OR) for both blood pressure and behavioral outcomes [35,36]. In most cases, blood pressure outcomes were grouped based on an SBP threshold of 140 mmHg and a DBP threshold of 90 mmHg (exceeding the threshold indicates poorly controlled blood pressure whereas values below the threshold indicate controlled blood pressure) unless stratification variables were applied and reported (eg, age or gender-specific thresholds, clinic vs remote measurements thresholds, multimorbidity thresholds). Behavioral outcomes were grouped based on the corresponding guidelines for health behavior change adopted by individual studies.

Change-from-baseline outcomes were calculated unless baseline data were missing, in which case changes at follow-up were included in the analysis. The random-effects meta-analysis method was used to account for the distribution of the effect across studies [37].

The *I*^2^ statistic was used to estimate the percentage of the variability in the effect estimates that is due to heterogeneity rather than chance [35]. Heterogeneity was explored further via subgroup analyses to investigate whether study-level variables could explain the observed heterogeneity.

Frequencies were used to summarize the behavioral strategies coded for each of the intervention and comparator groups [38]. Intervention strategies coded more than 3 times (frequency above 3) were considered for inclusion in the analysis. Subgroup analyses were performed to test for quantitative interactions, that is, whether intervention behavioral strategies could explain variation in the effect size.

Publication bias was examined by visual inspection of funnel plots and the Egger test. The meta-analysis was conducted using RevMan (version 5.4; The Cochrane Collaboration) [39].

## Results

### Overview

**Figure 1:**
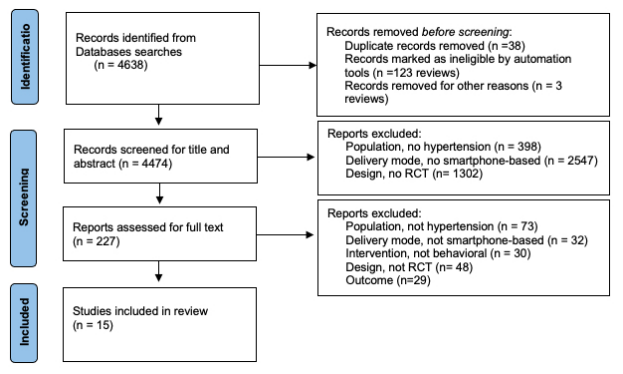
The PRISMA flowchart.

The systematic search of the 7 databases identified 4638 articles, of which 227 were included for full-text screening. One additional trial was identified from another source. A total of 15 randomized controlled trials with 7415 participants met all the eligibility criteria and were included in the analysis (Figure 1).

The majority of the included trials were conducted in the United States [40-45], whereas 2 studies were conducted in Australia [46,47], and 1 study in each of the following countries: Canada [48], China [49], New Zealand [50], Ghana [51], India [52], China and India [53], and Norway [54]. Participants (adults aged >18 years) were recruited from primary and secondary health care settings (Multimedia Appendix 2).

### Meta-analysis

#### Blood Pressure

**Figure 2:**
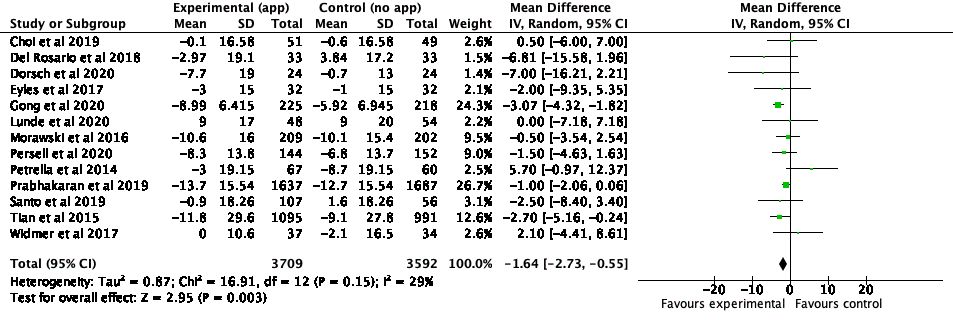
Meta-analysis of continuous outcome measurements for systolic blood pressure.

**Figure 3:**
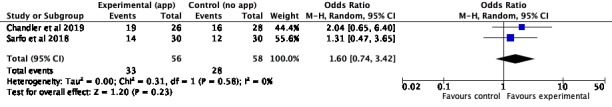
Meta-analysis of dichotomous outcome measurements for systolic blood pressure.

The meta-analysis suggested that behavioral self-monitoring interventions via smartphone apps have a small but significant effect on reducing SPB by an average of 1.64 mmHg (95% CI 2.73-0.55, n=7301; Figure 2) across the studies among those in the intervention group compared to those in the control group. A similar but insignificant effect was found among studies measuring changes in SBP based on recommended thresholds; participants receiving the intervention were, on average, 60% more likely to achieve recommended levels of SBP (eg, SBP below 140 mmHg for measurements obtained in clinic) compared to those in the control group (OR 1.60, 95% CI 0.74-3.42, n=114; Figure 3).

A similar direction of effect, though not significant, was found for the impact of the app-based behavioral self-monitoring interventions in changing DBP. The interventions had a small effect in changing DBP by an average of 0.39 mmHg (95% CI –2.01 to 1.23, n=1368; Multimedia Appendix 3) compared to the control. The effect of the intervention in supporting reductions in DBP (eg, DBP below 90 mmHg) was, on average, 41% more likely in the intervention than in the control (OR 1.41, 95% CI 0.66-3.01, n=114; Multimedia Appendix 4), though the changes were not different between the 2 groups.

Heterogeneity between studies was low for most blood pressure outcome measurements (SBP, continuous: *I*^2^=29%, Τ^2^=0.87, *P*=.15; SBP, dichotomous: *I*^2^=0%, Τ^2^=0, *P*=.58; DBP, continuous: *I*^2^=53%, Τ^2^=2.56, *P*=.04; DBP, dichotomous: *I*^2^=0%, Τ^2^=0, *P*=.54), suggesting that there is potentially small, unimportant variation in the effect beyond chance between the 2 studies that operationalized the blood pressure outcome using categorical thresholds.

#### Medication Adherence

The SMD between the intervention and control groups was medium to large (SMD 0.78, 95% CI 0.22-1.34, n=688; Multimedia Appendix 5), suggesting that app-based behavioral self-monitoring is significantly more effective at supporting improvements in medication adherence behavior compared to the control. A similar direction of effect was found for the subsample of studies that used categorical operationalization for the intervention effect and suggested those receiving the app-based behavioral self-monitoring intervention are, on average, 3.8 times more likely to achieve clinically meaningful medication adherence than those not receiving the intervention (OR 3.83, 95% CI 1.25-11.76, n=6428; Multimedia Appendix 6).

#### Physical Activity

The review found a moderate but insignificant effect of the app-based behavioral self-monitoring interventions in improving physical activity (SMD 1.63, 95% CI –0.35 to 0.87, n=501; Multimedia Appendix 7) although only 4 studies provided data for this, with one of the included studies suggesting that intervention group patients were 1.6 times more likely to adhere to the lifestyle change.

#### Diet

The meta-analysis included 4 studies on healthy diet and suggested a moderate effect of behavioral self-monitoring on changing dietary habits by reducing the consumption of high sodium food by an SMD of 0.44 (95% CI 0.08-0.79, n=382; Multimedia Appendix 8). Objective measures of urinalysis suggested a positive but insignificant trend of the behavioral intervention in reducing salt intake. Although promising, these results should be interpreted with caution due to the small number of studies and sample size contributing to the meta-analyses.

#### Smoking and Alcohol

One study found, on average, a 53% improvement in smoking cessation among those receiving an app-based self-monitoring intervention compared to those in the control group (OR 1.53, 95% CI 0.76-3.09, n=3698) [52]. Effects on alcohol consumption were very small and not significant.

### Subgroup Analyses

The most frequent behavior change technique coded in app-based behavioral self-monitoring interventions was feedback on behavior (n=13). Many app-based interventions (n=8) prompted participants to obtain advice from a health care provider following the behavioral measurements, and some (n=6) provided tailored advice to address the underlying mechanisms of behavior change. Goal setting of behavior, information about health consequences, and generic information about hypertension were strategies each coded in a small number of interventions (n=4). The most frequent strategy coded across both the intervention and control groups was reporting blood pressure and feedback on blood pressure (Multimedia Appendix 9).

Subgroup analysis found that tailored interventions resulted in higher and significant changes in both SBP and DBP in comparison to nontailored interventions (SBP: –2.92 mmHg, 95% CI –3.94 to –1.90, n=3102 vs –0.72 mmHg, 95% CI –1.67 to 0.23, n=4199, *χ*^2^=9.65, *P*=.002; DBP: –2.05 mmHg, 95% CI –3.10 to –1.01, n=968 vs 1.54 mmHg, 95% CI –0.53 to 3.61, n=400, *χ*^2^=9.19, *P*=.002). The differences between the 2 conditions were statistically significant and clinically meaningful (Multimedia Appendices 10 and 11).

Further investigation of the data revealed no effect of preselected variables that could influence blood pressure outcome (eg, sample size, time of follow-up, blood pressure outcome measurement obtained at clinic or remotely) on the observed effect.

### Risk of Bias

The risk-of-bias analyses suggested that studies were of low risk of bias. Inspection of funnel plots and the Egger test suggested a low risk of publication bias (Multimedia Appendix 12).

## Discussion

### Principal Findings

This systematic literature review and meta-analysis included 15 randomized controlled trials with 7415 participants and found that patients treated for hypertension receiving an app-based behavioral self-monitoring intervention reduced SBP by an average of 1.64 mmHg (95% CI 2.73-0.55) and were, on average, 60% more likely to reduce SBP to <140 mmHg and DBP to <90 mmHg compared to those in the control group. Further subgroup analysis suggested that behavioral self-monitoring interventions combined with tailored advice had a higher and potentially clinically meaningful effect on reducing both SBP (mean reduction of 2.92 mmHg) and DBP (mean reduction of 2.05 mmHg) [55,56].

This study found that app-based self-monitoring of behavior interventions increased the odds of achieving medication adherence by 3 folds in the intervention group compared to the control. The significant effect of the app-based behavioral self-monitoring interventions in supporting improvements in both blood pressure and medication adherence provides us with confidence that such interventions could be effective solutions to support health behavior change and thus reduce blood pressure in patients treated for hypertension during blood pressure checks or similar clinical consultations.

The behavioral interventions indicated positive effects for supporting improvements in healthy diet by reducing the consumption of high sodium food, as well as positive trends in supporting physical activity, smoking cessation, and alcohol consumption. Although promising, a small number of studies contributed to these meta-analyses, and thus the results should be treated with caution.

### Strengths and Limitations

This review has several strengths and limitations. It did not include gray literature or unpublished studies and was limited to searching a few publicly accessible databases only. Nevertheless, this review summarizes the currently available evidence and suggests that behavioral tailored self-monitoring interventions are effective in changing SBP and DBP by –2.92 mmHg and –2.05 mmHg on average, respectively, compared to usual care, enhanced usual care, or a minimal behavioral intervention.

A limitation of the included studies is the use of self-reported measurements for the behavioral outcomes, which are inherent to bias. This might have diminished the validity of the observed intervention effect on health behaviors. Future trials should employ valid methods of measurement to assess behavioral outcomes and thus inform recommendations for policy making and practice.

This review has evaluated randomized controlled trials that compared behavioral self-monitoring interventions with usual care, enhanced usual care, or minimal behavioral interventions. We have used an extensive search strategy and identified all publicly available evidence. We have adopted a rigorous approach to data extraction and intervention coding to generate the results and form recommendations for best practices and future intervention development.

### Implications for Practice and Intervention Development

The included trials had a duration of 1 to 12 months; thus, the evidence for the sustained effects of the intervention remains uncertain. However, a limited number of studies with long-term measurements had positive trends toward blood pressure reduction. Considering the wide reach and low-cost use of mobile technologies, this evidence indicates the potential impact of behavioral interventions on overall hypertension-related morbidity and mortality.

The comparator group included usual care (eg, clinic blood pressure checks), enhanced usual care (eg, regular blood pressure checks and medication adjustments), or minimal generic lifestyle interventions (eg, lifestyle tips and advice), suggesting that tailored behavioral self-monitoring is an acceptable addition to usual care and has a small, though clinically meaningful, effect on reducing blood pressure beyond and above usual care clinical practice.

Many studies involved clinicians in signposting participants to the app-based behavioral intervention, which might have influenced participants’ engagement with the intervention and their health care. Moreover, the most frequent strategies reported being used with the behavioral self-monitoring interventions were feedback on health behaviors and prompts to obtain advice from a health care provider following behavioral measurements that require further support and monitoring. Although none of these strategies individually explained clinical effectiveness, they could have a synergistic effect in supporting patients’ engagement with self-monitoring processes and thus in generating the observed improvements in health behaviors and reductions in blood pressure.

However, due to the limited information reported by primary studies, this review could not provide comprehensive, theory-based evidence on the mechanism by which the self-monitoring intervention achieved the observed clinical effectiveness [23-26]. Only a small number of studies explicitly reported on the theoretical concepts that informed the health behavior change intervention. For example, Chandler et al [40] and Dorsch et al [42] reported that the intervention aimed to modify beliefs and attitudes to support self-regulation processes and bring about changes in health behaviors. However, there is no evidence on the effects of the interventions with regard to modifying these theoretical influences to achieve changes in health behaviors and blood pressure. It would be useful if future studies report on the theoretical underpinnings and use valid measurements of engagement with the intervention strategies, as well as the underpinnings of health behaviors, to facilitate the generation of rigorous and replicable evidence on the mechanisms by which self-monitoring of behavior via the use of digital interventions support health behavior change and clinical effectiveness [57].

### Conclusion

This systematic literature review suggested that tailored behavioral self-monitoring of hypertension-related behaviors facilitated via smartphone apps is effective in reducing blood pressure by an average of 2 mmHg above and beyond usual care, enhanced usual care, or minimal behavioral interventions. Thus, clinical practice should recommend behavioral self-monitoring combined with tailored behavioral advice to achieve clinical effectiveness. Considering the wide use of smartphone apps and their potential to reach large numbers of people, app-based behavioral self-monitoring interventions combined with tailored behavioral advice could potentially be a cost-effective addition to usual care blood pressure consultations. However, due to the limited quality of the trials included in this review, future research with rigorous methods is required to determine the direct impact of such interventions on both health behavior change and blood pressure, as well as their indirect effects on service provision and hypertension-related morbidity and mortality.

## Appendix

Multimedia Appendix 1 Search strategy for MEDLINE.

Multimedia Appendix 2 Study characteristics.

Multimedia Appendix 3 Meta-analysis of continuous outcome measurements for diastolic blood pressure.

Multimedia Appendix 4 Meta-analysis of dichotomous outcome measurements for diastolic blood pressure.

Multimedia Appendix 5 Meta-analysis of continuous outcome measurements for medication adherence.

Multimedia Appendix 6 Meta-analysis of dichotomous outcome measurements for medication adherence.

Multimedia Appendix 7 Meta-analysis of continuous outcome measurements for physical activity.

Multimedia Appendix 8 Meta-analysis of continuous outcome measurements for healthy diet (consumption of low sodium food).

Multimedia Appendix 9 Intervention coding of experimental and comparator groups.

Multimedia Appendix 10 Subgroup analysis for the systolic blood pressure continuous outcome.

Multimedia Appendix 11 Subgroup analysis for the diastolic blood pressure dichotomous outcome.

Multimedia Appendix 12 Funnel plot of the systolic blood pressure continuous outcome.

Multimedia Appendix 13 The PRISMA (Preferred Reporting Items for Systematic Reviews and Meta-Analyses) checklist.

## Abbreviations

CENTRAL: Cochrane Central Register of Controlled Trials
DBP: diastolic blood pressure
NIHR: National Institute for Health and Care Research
OR: odds ratio
SBP: systolic blood pressure
SMD: standardized mean difference

## Data Availability

All data included in this review are reported in the multimedia appendices.
