# Supplementary material for: The Association Between Smartphone App–Based Self-monitoring of Hypertension-Related Behaviors and Reductions in High Blood Pressure: Systematic Review and Meta-analysis
Source: JMIR Mhealth Uhealth. 2022 Jul 12;10(7):e34767. doi: 10.2196/34767 (PMC9328789; doi:10.2196/34767)
Supplement: Multimedia Appendix 1 [file mhealth_v10i7e34767_app1.docx]

**Multimedia Appendix 1**

Table 1. Search strategy for MEDLINE

| **MEDLINE via PubMed**  smartphone OR cell phone OR cellphone OR mobile phone OR app OR apps OR application* OR mhealth OR mobile health OR ehealth OR e-health OR technolog* OR online  AND  diastolic pressure OR pulse pressure OR systolic pressure OR blood pressure OR hypertension OR hypertension [MeSH Terms]  AND  behavior* OR behaviour* OR smok* OR medic* adherence OR medic* concordan* OR medic* complian* OR physical* activ* OR exercis* OR alcohol* OR nutrition* OR diet* OR *weigh*  OR smoking[MeSH Terms] OR smoking cessation[MeSH Terms] OR medication adherence[MeSH Terms] OR exercise[MeSH Terms] OR alcohol drinking[MeSH Terms] OR alcohol abstinence[MeSH Terms] OR diet[MeSH Terms]  AND  trial* OR rct OR random* OR placebo* OR blind* OR control* |
| --- |
