# Supplementary material for: The Association Between Smartphone App–Based Self-monitoring of Hypertension-Related Behaviors and Reductions in High Blood Pressure: Systematic Review and Meta-analysis
Source: JMIR Mhealth Uhealth. 2022 Jul 12;10(7):e34767. doi: 10.2196/34767 (PMC9328789; doi:10.2196/34767)
Supplement: Multimedia Appendix 2 [file mhealth_v10i7e34767_app2.docx]

**Multimedia Appendix 2**

**Table 2**. Study Characteristics

| **Study, Country, Design** | **Recruitment** | **Intervention** | **Control** | **Outcomes and measurement** |
| --- | --- | --- | --- | --- |
| Chandler et al., 2019  USA  RCT | Patients contacted via phone and booked in for initial BP screening assessment | Smartphone Medication Adherence Stops Hypertension (SMASH), Bluetooth-enabled BP monitor and an electronic medication tray | Text messages including links to PDFs and brief video clips containing healthy lifestyle tips for attention control | Follow up at 1,6 and 9 months  Blood pressure: proportion of participants meeting the recommended thresholds for controlled SBP and DBP, change from baseline to 6 months. SBP [ I 19/26 C 16/28] and DPB [ I 11/26 C 8/28] and mean change from baseline to follow up (not estimable); measurement obtained by ambulatory BP readings  Medication adherence: self-reported mean difference at 6 months [ I 9.81 (1.31) 26 C 6.84 (1.52) 28]; measurements obtained by questionnaire |
| Choi et al., 2019  USA  RCT | Recruited from the cardiology clinic of an academic medical centre | Smartphone app for duration of 6 months | Counselling at 1 and 3 months | Follow up at 1, 3 and 6 months  Blood pressure: mean change from baseline to 6 months SBP [ I -0.1 (16.58) 51 C -0.6 (16.58) 49] and DBP [ I -0.4 (9.95) 51 C -1 (9.95) 49]; measured at clinic by RD  Diet: mean change of Mediterranean diet from baseline to follow up (not estimable) and proportion of participants meeting guidelines for high compliance with the Mediterranean diet, change from baseline to follow up (not estimable); measured obtained by self-reported 14-items of adherences to Mediterranean diet |
| Del Rosario et al., 2018  Australia  RCT | Patients referred to hospital for cardiac-related diagnosis were recruited | The Smartphone Technology and Heart Rehabilitation (STAHR) app | No additional advice | Follow up at 6 weeks  Blood pressure: mean change from baseline to 6 weeks SBP [ I -2.97 (19.10) 33 C 3.84 (17.2) 33] and DBP [ I -0.4 (9.95) 51 C -1.88 (10.1) 33]; measurement obtained at clinic  Physical activity: six-minute walking distance (a clinically established metric to evaluate sub-maximal exercise capacity in CRPs) correlated with the average walking time/day captured at mobile phone (not estimable) |
| Dorsch et al 2020  USA  RCT | Patients recruited though the university health centre recruitment platform and by sending letters | Smartphone app with geo tracking | No additional advice | Baseline and 8 weeks  Blood pressure: mean change over the 8 weeks in SBP [ I -7.7 (19) 24 C -0.7 (13) 24 ]; measured bi-weekly by the participants using their own home blood pressure monitors  Diet: mean change in sodium excretion from baseline to 8 weeks [ I -462 (1220) 24 C -382 (1460) 24] ^a^; measures by urinary excretion of sodium by the a urine sample  mean change in sodium [ I -1537 (2693) 24 C -233 (2150) 24]; measured by self-reported 24-hour dietary assessment tool (ASA24)  mean change in sodium [ I -1553 (1764) 24 C -515 (1081) 24]; measured by sodium screener survey to estimate the quantity and frequency of consumption of high-sodium food in participants reports |
| Eyles et al., 2017  New Zealand  RCT | Adverts at supermarkets, hospital, healthcare organization newsletters, and social media; newspapers; posters at clinics. | SaltSwitch smartphone app for 4 weeks | Access to cardiac rehabilitation services as per usual care for people with CVD in New Zealand | Follow up at 4 weeks  Blood pressure: change from baseline to 4 weeks SBP [ I -3 (15) 32 C -1 (15) 32] and DBP (not estimable); blood pressure measurement at the university  Diet, salt consumption: mean change of salt consumption from baseline to 4 weeks [ I 0.7 g/MJ (0.52) 33 C 1.0 g/MJ (0.58) 33]^a^ ; measurement obtained by household food purchases salt content of household food purchases (g/MJ)  Urinary sodium: mean between group difference at sodium at 4 weeks follow up [ I 3534 (536) 32 C 3545 (536) 32] measurement obtained by a random (spot) urine sample. Spot urine concentration was converted into an estimate of 24-hour sodium excretion using the INTERSALT formula |
| Gong et al., 2020  China  RCT | Patients diagnosed with primary hypertension were enrolled from 38 hospitals | Yan Fu app | Usual care | Follow up every month, 6 months  Blood pressure: mean change from baseline to 6 months [ SBP I -8.99 (6.41) 225 C -5.92 (6.94) 218] and DBP [ SBP I – 7.04 (6.13) 225 C -4.24 (8.13) 218]^a^ and mean change in percentage of participants with controlled BP; baseline measurement obtained at clinic follow up using ambulatory blood pressure monitors (for control) or app (for intervention)  Medication Adherence: mean change from baseline to 6 months [I 48/225 C 10/218] proportion of medium and high adherence; measurement obtained by 8 item self-report |
| Lunde et al., 2020  RCT  Norway | Patients were recruited from two cardiac rehabilitation centres | Smartphone app | Usual care | Follow up at 12 months  Blood pressure: mean change from baseline to 12 months [ SBP I 9 (17) 48 C 9 (20) 54] and DBP [ o 5 (11) 48 C 3 (10) 54]; measurement obtained at clinic  Exercise habits: mean change from baseline to 12 months [ I 1.4 (1.5) 48 C 0.6 (1.1) 54] Exercise habits were defined as mean exercise sessions taken each week for the last year. In this context, an exercise session was defined as structured activity lasting at least 30 min, where you got both sweaty and breathless, and felt like taking a shower afterwards; measurement obtained by self-reports |
| Morawski et al., 2018  USA  RCT | Online patient communities, social media, pertinent mobile apps, and targeted adverts. | Medisafe app | Usual care | Follow up at 4, 8 and 12 weeks  Blood pressure: mean change from baseline to 12 months SBP [ I -10.6 (16.0) 209 C -10.1 (15.4) 202] and DBP (not estimable); measurement was obtained participants in both treatment groups are contacted and asked to check their blood pressure using the Bluetooth-enabled blood pressure cuff that they were provided at enrolment. Blood pressure is assessed as the average of 2 measurements, taken at least 5 minutes apart  Medication adherence: mean change from baseline to 12 weeks follow up [ I 0.4 (1.5) 209 C -0.01 (1.5) 202]; measurement obtained by self-reported 8 items questionnaire |
| Persell et al., 2020  USA  RCT | Patients were recruited from outpatient primary care clinics | Mobile phone-based tracking application | Enhanced usual care: remote BP measurements | Follow up at 6 months  Blood pressure: mean change from baseline to 6 months SBP [ I -8.3 (13.8) 144 C 6.8 (13.7) 154] and DBP −4.3 (8.4) 144 −3.6 (9.5) 152]; BP measurement at clinic by research staff  Medication adherence: mean change from baseline in proportion of participants fully adherent [ I 2/108 C 0/93]; measurement obtained by self-reported 4-day recall  Diet: mean change [ I 1.1 (12) 108 C 1.9 (12.08) 93] days per week eating several other food categories (processed meat, fried food, sugar, baked goods or ice cream); measurement obtained by Dietary Approaches to Stop Hypertension diet compliance Questionnaire  Physical activity: mean change [ I 16 (2.9) 93 C 5.1 (0.4) 108] minutes per week of at-least-moderate physical activity; measurement obtained by self-reports |
| Petrella et al., 2014  Canada  RCT | Patients were recruited via adverts printed or radio, word of mouth, community presentations and physician referral. | Tailored exercise program and health monitoring app | Tailored exercise program (similar to intervention group) | Follow up at 12 weeks  Blood pressure: change from baseline to 12 weeks SBP [ I -3 (19.15) 67 C - 8.7 (19.15) 60] and DBP [ I -2.5 (10.2) 67 C – 4.9 (10.2) 60] ; measurement obtained at clinic  Physical activity: between group difference in means at 12 week follow up [ I 188.2 (189.5) 67 C 170.3 (161.2) 60] minutes of exercise per week; measured by self-reports: participants logged pedometer-based compliance to exercise at the app (experimental) or paper (control). Four measurements in total, one corresponding to each time point, were included as part of the outcome variable; [ I 27/67 C 18/60 ] had high compliance to the recommendation of 150min of exercise per week. |
| Prabhakaran et al., 2019  India  Cluster RCT | Patients were recruited by nurses at 20 community health centres | mWellcare system | Enhanced usual care | Follow up at 12 months  Blood pressure: mean change from baseline to 12 months SBP [ I -13.7 (15.5) 1842 C -12.7 (15.5) 1856] and DBP [ I -6.5 (10.5) 1842 C -9.5 (10.5) 1856] ; measurement obtained at clinic  Tobacco use: change in tobacco use from baseline to 12 months [ I -6% reduction in tobacco users 19.5/1842 C -7% reduction in tobacco users 12.8/1856] number of participants using tobacco; measurement obtained by self-report  Alcohol use: mean change from baseline to follow up [ I -3.8% mean reduction; 8.7/1842 alcohol users C -2.4% reduction; 0.3/1856 in alcohol users]; measurement obtained using the Alcohol Use Disorder Identification Test  Medication adherence: between group difference in days of adherence during the past week at 12 months follow up [ I 648 / 1856 C 833/1842]; measurement obtained by self-report |
| Santo et al., 2018  Australia  RCT | Patients with coronary health diseases from a tertiary hospital | Enhanced app | Usual care | Follow up at 3 months  Blood pressure: mean change from baseline to 3 months SBP [ I -0.9 (18.26) 107 C 1.6 (18.26) 56] and DBP [ I -0.1 (11.42) 107 C 2.2 (10.51) 56] ; measurement obtained at clinic  Medication adherence: mean change from baseline to 3 months [ I 0.31 (1.4) 107 C -0.17 (1.26) 56]; measurement obtained by self-reported 8 items questionnaire |
| Sarfo et al., 2019  Ghana  RCT | Outpatient Neurology clinic at Teaching Hospital | smartphone with an App for 3 months | Usual care | Follow up at 9 months  Blood pressure: between group difference at 9 months follow up SBP [ I 14/30 C 12/30] and DBP [ I 14/30 C 12/30]; measurement obtained at clinic  Medication adherence: mean change from baseline to 9 months follow up [ I 2.7 (1.5) 30 C 1.5 (1.7) 30] ^a^ ; measured by self-reported 8 items questionnaire. Medication possession ratio [ I 22/30 C 22/30] at 9months follow up, measurement obtained by refill prescription data at 3,5 and 9 months |
| Tian et al., 2015  China and India  Cluster RCT | Conducted in 47 villages (27 in China and 20 in India). 2,086 patients | Android-powered “app” | Standard care | Follow up at 12 months  Blood Pressure: mean change from baseline to 12 months follow up SBP [ I -11.8 (29.6) 1095) C -9.1 (27.8) 991] and DBP (not estimable); measurement obtained at clinic  Medication adherence. Mean change of adherent participant [ I 351/1095 C 65/991]; measured by self-reports |
| Widmer et al., 2017  USA  RCT | Patients were recruited, consented, and enrolled in a prospective fashion after PCI according to an approved Mayo Clinic IRB protocol | Digital health intervention | Usual care | Follow up at 3 months  Blood pressure: mean change from baseline to 3 months SBP [ I (10.6) 37 C -2.1 (16.5) 34] and DBP [ I -4.0 (11.9) 37 C -4.4 (13.1) 34] ; measurements obtained at clinic  Diet: scores were calculated by the summation of daily servings of fruits, vegetables, whole grains, and lean proteins with points taken away for daily servings of saturated fats and sweets [ I 1.4 (3.2) 34 C 4.1 (4.1) 34]; measurement obtained by self-reports  Physical activity: mean change of minutes exercises per week [ I 179 (109.1) 37 C 139 (87.7) 34 ]; measured using self-reported questionnaires  Medication adherence: (not estimable) measurement obtained by self- reports  Smoking: (not estimable) measurement obtained by self- reports |

^a^ data included in meta-analysis

I= intervention group, C=control group. Values are reported as means (SD) or number/total
