# Supplementary material for: The Association Between Smartphone App–Based Self-monitoring of Hypertension-Related Behaviors and Reductions in High Blood Pressure: Systematic Review and Meta-analysis
Source: JMIR Mhealth Uhealth. 2022 Jul 12;10(7):e34767. doi: 10.2196/34767 (PMC9328789; doi:10.2196/34767)
Supplement: Multimedia Appendix 3 [file mhealth_v10i7e34767_app3.docx]

**Multimedia Appendix 3. Meta-analysis of continuous outcome measurements for diastolic blood pressure.**


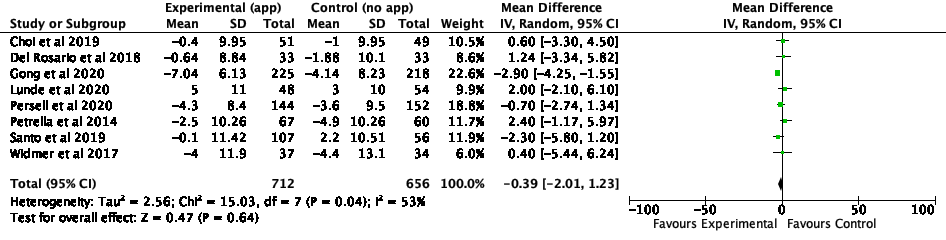


**Figure 1**. Meta-analysis of continuous outcome measurements for Diastolic Blood Pressure
