# Supplementary material for: The Association Between Smartphone App–Based Self-monitoring of Hypertension-Related Behaviors and Reductions in High Blood Pressure: Systematic Review and Meta-analysis
Source: JMIR Mhealth Uhealth. 2022 Jul 12;10(7):e34767. doi: 10.2196/34767 (PMC9328789; doi:10.2196/34767)
Supplement: Multimedia Appendix 8 [file mhealth_v10i7e34767_app8.docx]

**Multimedia Appendix 8. Meta-analysis of continuous outcome measurements for healthy diet (consumption of low sodium food).**

**
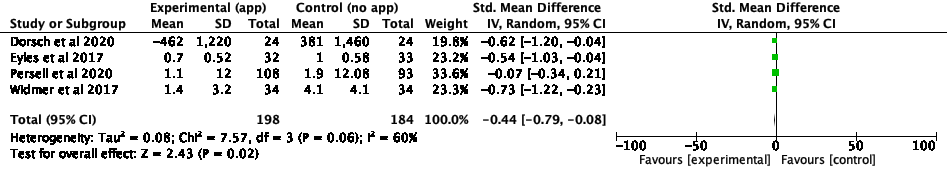
**

**Figure 6.** Meta-analysis of continuous outcome measurements for Healthy Diet (reduce consumption of high-sodium food)
