# Supplementary material for: The Association Between Smartphone App–Based Self-monitoring of Hypertension-Related Behaviors and Reductions in High Blood Pressure: Systematic Review and Meta-analysis
Source: JMIR Mhealth Uhealth. 2022 Jul 12;10(7):e34767. doi: 10.2196/34767 (PMC9328789; doi:10.2196/34767)
Supplement: Multimedia Appendix 9 [file mhealth_v10i7e34767_app9.docx]

**Multimedia Appendix 9. Intervention coding of experimental and comparator groups.**

| Study | **Experimental Group** | | Comparator Group |
| --- | --- | --- | --- |
|  | Monitoring Health Behaviour | Additional intervention strategies and mechanisms |  |
| Chandler et al., 2019 | Medication adherence  *Medication trays provided a series of reminder signals to take medications and medication taking is uploaded at the app* | Tailored  *These messages were based upon the participant’s responses to a branch logic questionnaire, which identified their values, beliefs, and short/long term life goals*  Theoretical underpinnings  *Association of one’s increasing MA being linked to their core values, beliefs, and life goals, ultimately fostering greater sustained engagement via increased autonomous motivation to adhere to the regimen*  2.7. Feedback on BP  *the user received audio and visual feedback of their systolic and diastolic BP and heart rate levels. The app also provided a cumulative table of average BPs displayed in categories of daily, weekly, and/or monthly BP progress reports*  2.2. Feedback on behaviour  *automated tailored motivational/social reinforcement messages based upon MA levels each participant’s previous day’s adherence score. Initially, the frequency of SMS messages received was based on the previous day’s MA score. After 4 weeks of 100% MA, message delivery changed to a 3-day interval schedule*  Blood pressure monitoring  *Participant was to measure their BPs every three days using the Bluetooth monitor paired with the SMASH app.* | 3.1. Social Support Unspecified  *Minimal intervention: text messages including links to PDFs and brief video clips containing healthy lifestyle tips* |
| Choi et al., 2019 | Diet  *Participants were encouraged to use the app to take pictures of their food, document meals and amounts consumed, ask questions to the RD*  *The app included weekly challenges to encourage dietary modification*  Physical activity  *Participants were encouraged to use the app to document exercise* | 1.1. Goal setting behaviour  8.7 Graded tasks  *The challenges were for patients to challenge themselves (i.e., they did not compete against other participants). Examples of challenges included increasing daily servings of vegetables or exercising daily*  2.2. Feedback on behaviour  *Participants received the RD’s feedback directly to their mobile phone to review at their own convenience*  1.5 Review behavioural goals  *Participants in EXP received dietary counselling from their RD via the app in the context of their meal logs*  3.1. Social support unspecified  either the RD or the patient could initiate contact through the app  Blood Pressure monitoring  *Participants were encouraged to monitor their BP and track BP in the log* | Two 30 min counselling sessions by RD: at 1 and 3 months  1.5. Review behavioural goals  *RD reviewed dietary recall*  3.1. Social support unspecified  *education reinforced and strategies for improvement developed* |
| Del Rosario et al., 2018 | Physical activity  *the app an objective estimate of physical activity estimated by a smartphone-based algorithm that utilized the measurements from the smartphone’s inertial measurement unit, and digital altimeter. Participants were instructed to turn on the smartphone each morning and place it in their pants pocket during the waking day, and recharge the device every night before going to sleep (walking time/day)* | Tailored  2.2. Feedback on physical activity behaviour  1.6 Discrepancy between current behaviour physical activity and goal  *You have not walked for more than 30 minutes for the last # days*  2.7. Feedback on BP  2.7. Feedback on weight  Blood Pressure monitoring  *The app leverages NFC technology, that enabled the measurements to be wirelessly transmitted to the SG3 via Android’s NFC-based ‘Tap-And- Go’ eliminating the need for blood pressure and weight to be input manually*  Weight  *NFC enabled digital weight scale that enabled the measurements to be wirelessly transmitted to the SG3 via Android’s NFC-based ‘Tap-And-Go.’* | 3.1. Social support unspecified  *Standard outpatient hospital-based CRP* |
| Dorsch et al., 2020 | Diet  *The patient scans foods and obtain information about the sodium*  *Daily push notifications will prompt patients to document if they have reduced those foods from their diet* | Tailored  Location based notifications to provide dietary recommendations  Theoretical underpinnings  Recommendations are informed by beliefs and attitudes based on theory of planned behaviour, self-regulation, and mindful decision making  4.2 information about antecedents (education)  2.7 Feedback on behaviour  5.1. Information about health consequences | No additional advice |
| Eyles et al., 2017 | Diet  *SaltSwitch enables users to make lower salt food choices by scanning the barcode of a packaged food using their smartphone camera and to receive an immediate interpretive, traffic light nutrition label on screen, along with a list of lower salt alternatives to ‘switch’ to. When a user scans the barcode of a product not already in the database, they are prompted to take three photographs (one each for the front of the pack, the nutrition information panel, and the ingredient list). Photographs are then sent to the research team and new products are subsequently added to the app. Weekly reminder text messages were sent to intervention participants to encourage them to use the app when shopping for household food and beverages to record purchase of products with salt, as a proxy of salt intake* | 5.1. information about health consequences  4.2 information about antecedents (education)  *The traffic light labels are determined using the United Kingdom’s Guidance for creating a front of pack nutrition label for pre-packaged foods, and healthier options are determined using the Australia New Zealand Food Standards Agency Nutrient Profiling Scoring Calculator (NPSC), a nutrient profiling model (scoring system to rank foods) used to determine whether foods are eligible to carry nutrition and health claims in Australia and New Zealand. The NPSC system allocates a score to food products by balancing positive nutrients such as fibre and protein with negative nutrients including salt and saturated fat* | No additional advice |
| Gong et al., 2020 | Medication adherence  *The participants were instructed to record if they had taken medicine in the app* | Tailored advice  *Provides 4 kinds of health evaluations and gives personal management advice*  7.1 Prompts/cues  *Reminders cues to take medicine and exercise*  *The app would remind the participants about the dose and timing of their drugs*  3.1. Social support unspecified \| education  *Users can query information about hypertension through the app*  *Users can have remote consultation with professional doctors*  2.1. Monitoring of behaviours by others without feedback  *Relatives and doctors can monitor the health records of the participant*  Blood pressure monitoring  *The participants were instructed to upload their blood pressure at least once daily in the app. Bluetooth between the app and the automatic sphygmomanometer, which was provided by the Shanghai O2O Care Company.*  *If the participants did not upload the blood pressure measurements or medication record, the app would send messages to remind them*  *If the blood pressure was abnormal, the app would warn participants to measure their blood pressure again after taking a rest and remind* *them it is time to see the doctor* | Blood pressure monitoring  *patients in the control group did not use any hypertension management applications but they needed to measure their blood pressure using the same sphygmomanometer and record their blood pressure on paper every day* |
| Lunde et al., 2020 | Physical activity  *The patients replied with a red or green face, depending on whether they had completed the planned task or not, and rated their weekly goal achievement on a scale from 0 to 100* | - 1. Goal setting (behaviour)   *The app permitted the patient to set individual goals with tasks and accompanying reminders*  *Goals and tasks decided during baseline assessment were added to the app, and each patient decided when and how often reminders of their tasks should appear*  7.1. Prompts and cues  *The app itself provided automatic reminders and evaluations of tasks and weekly goal achievement*  2.2. Feedback on physical activity, app  *During the follow-up period, the patients received short, tailored, individualized motivational feedback directly through the app 1–3 times a week. Additionally, they received comprehensive individual feedback via email once a week for the first 12 weeks and every fourth week for the rest of the year. All feedback was based on what each patient had done, not done or on each patient’s notes*  3.1. Social support unspecified, HCP  2.2. Feedback on physical activity, HCP  *A supervisor had access to an administrator interface and monitored the goals, tasks and notes of each patient in the IG. Patients could submit questions to the supervisor at any time and would receive an answer within two working days. Patients were followed for one year by the same supervisor who included the patients at baseline. The same supervisor monitored and gave feedback to all patients in the IG for the whole year. The supervisor was a physiotherapist specialized in cardiovascular and pulmonary physiotherapy with seven years of experience in CR. A more detailed description of the intervention has been previously reported by our group* |  |
| Morawski et al., 2018 | Medication adherence  *Reporting medication taking as response to app notifications* | 8.3 Habit formation  *It provides alerts to patients when it is time to take medication*    2.2. Feedback on medication adherence  *Medisafe also allows patients to generate weekly reports of medication adherence*  2.7. Feedback on BP   - 1. Goal setting (behaviour)   *Medication lists can be entered manually, along with timing of administration as recommended by their doctor or be auto populated through a linkage with an existing record in those cases where this integration has been established.*  3.1 Social support unspecified  *designate a “Medfriend” of their choosing, who is granted access to a patient's medication taking history, receives alerts when doses are missed, and can provide peer support*  Blood pressure monitoring  *The app allows patients to monitor biometric measurements (either directly into the application or through synchronization with the smartphone's non-Medisafe health devices)* | Blood Pressure monitoring  *remote measurement at 4,8 and 12 weeks and report measurements* |
| Persell et al., 2018 | Medication adherence  *The HPCP prompts medication users to set up medication reminders for each prescribed medication and then will check-in every month to ask about adherence*  Diet  *Coaching around healthy eating includes educational content and opportunities to log food and beverage consumption. Participants were prompted multiple times per day to log meals and snacks*  Physical activity  *Users can set physical activity goals and receive coaching on them, and activity is tracked via data collected from the iPhone's motion detector or the user may enter them manually. Participants are prompted multiple times per day to remind them to log physical activity* | Tailored  2.7. Feedback on BP  *The HPCP informs users if they have changes in their blood pressure over time based on a set of algorithms and provides appropriate coaching. For example, this can include a focus on improving medication adherence if the trend is upward, and, if the trend is downward, positive feedback that directly ties into participants' recorded health behaviours. Additionally, participants receive weekly and monthly feedback showing their overall trend and whether they are above or below their goal*  2.2. Feedback on medication adherence  *This feedback also includes behavioural and educational coaching and encourages the participants to check with their medical providers if blood pressure remains above 130 mmHg systolic or 80 mmHg diastolic for over one month*  7.1. Prompts and cues  *The HPCP prompts medication users to set up medication reminders for each prescribed medication and then will check-in every month to ask about adherence or any changes in the user's medication plan, providing coaching around barriers to adherence*  3.1 Social support unspecified  *It also prompts participants to contact their medical providers if certain barriers, such as side effects, are reported*  5.1. information about health consequences  4.2 information about antecedents  *Over time, the HPCP provides education on several hypertension-related topics. This includes coaching about weight loss and its relationship to hypertension. Coaching around healthy eating includes educational content and opportunities to log food and beverage consumption. The HPCP encourages users to choose a diet more consistent with the Dietary Approaches to Stop. Provides education on topics such as why self-monitoring and medication adherence are important in blood pressure management, how better blood pressure control can lower the risk of complications, and how users can increase their control over blood pressure with healthy behaviour changes*  2.2. Feedback on diet and physical activity  *The HPCP also provides coaching about physical activity. Users can set physical activity goals and receive coaching on them, and activity is tracked via data collected from the iPhone's motion detector or the user may enter them manually. The HPCP tracks sleep and provides coaching based on the user's sleep habits, and also includes education on stress management. Users can set goals and receive guidance and feedback through the app. They receive feedback immediately after data entry and in daily and weekly summary conversations. The HPCP sends messages multiple times per day to users to remind them to log blood pressure, medications, weight, meals and snacks, and physical activity, and users can initiate conversations with the app anytime. Content is customized based on the user's values and inputs.*  *The app does not send messages to participants' clinical care teams*  Blood pressure monitoring  *The HPCP promotes home blood pressure measurement through encouragement, reminders, and tracking, and it communicates to users their individual measurement values. The HPCP prompts users to retake readings if outlier values are recorded and prompts them to call their medical professional for extreme values (systolic ≥180 or <90 mmHg and/or diastolic ≥110 or<60 mmHg)* | Blood pressure monitoring  *Enhanced usual care: control group participants receive a home blood pressure monitoring device, are instructed on how to perform self-monitoring, and are asked to demonstrate the use of the device at the baseline study visit. Study staff also provide participants with information sheets describing home monitoring techniques and information on interpreting home readings. At the baseline visit, participants are assisted with the installation of an Omron application to their smartphone device that can be used to track HBPM data but does not provide interactive feedback. They are asked to demonstrate how to transmit data from the HBPM to their smartphone.* |
| Petrella et al., 2014 | Physical activity  *The home-monitoring protocol required participants to exercise daily and report pedometer steps three times per week* | 2.7. Feedback on BP  *Fig 1*  2.2. Feedback on physical activity  *Fig 1*  Blood pressure monitoring  *The home-monitoring protocol required participants to measure blood*  *pressure three times per week. When measurements were outside of pre-determined safety limits, an automated alert was sent to the study physician’s smartphone for follow up* | Tailored exercise program (similar to intervention group)  Physical activity logged at paper journal |
| Prabhakaran et al., 2019 | Tobacco use  *In the mWellcare arm, the mWellcare system collected data on tobacco use*  Alcohol  *In the mWellcare arm, the mWellcare system collected data on alcohol use*  Medication adherence  *In the mWellcare arm, the mWellcare system collected data on current medications.* | 7.1. Prompts and cues  *It was also equipped to send short message service reminders to medication adherence*  Blood Pressure measurement  *In the mWellcare arm, the mWellcare system collected data on blood pressure.* | Minimal intervention: at baseline NCD nurses provided and explained the lifestyle advice pamphlet (in local languages, Hindi and Kannada) for each participant. No additional advice or availability by nurse |
| Santo et al., 2018 | Medication adherence  *The app provided interactive and customisable features with daily reminders about medication taking, which could be snoozed, rescheduled and/or marked as a ‘taken’ or ‘missed’ dose* | Tailored   - 1. Goal setting (behaviour)   *Participants allocated to the interventions received instructions and assistance to download an app on their smartphones, input their current list of cardiovascular medications and set daily reminders at the time when each medication was usually taken*  8.3 Habit formation  *The app provided simple daily reminders, similar to an alarm or text message, to prompt the participants to take their medications at the correct time every day*  2.2. Feedback on medication adherence  *medication-related features including adherence statistics*  3.1 Social support unspecified  *ability to export and share information with others and ability to alert other people, such as family members, if the participant missed a medication dose* | Usual care |
| Sarfo et al., 2019 | Medication adherence  *Patients in the IG received a Blue-toothed UA-767Plus BT BP device and a smartphone with embedded application for monitoring and reporting medication intake under nurse guidance for three months* | 2.2. Feedback on medication adherence  *Tailored motivational text messages were delivered based upon levels of adherence to the medication intake regimen. A secure web page hosted at the Medical University of South Carolina automatically calculated the daily medication intake adherence scores*  2.7. Feedback on BP  *Tailored motivational text messages were delivered based upon levels of adherence to the medication intake regimen. A secure web page hosted at the Medical University of South Carolina automatically calculated the mean BP for each monitoring session*  Blood pressure monitoring  *Patients in the IG received a Blue-toothed UA-767Plus BT BP device and a smartphone with embedded application for monitoring and reporting BP measurements under nurse guidance for three months* | Text messages for generic lifestyle |
| Tian et al., 2015 | Medication adherence  *It consisted of prompts for patients to report on current medication use* | Tailored advice, by HCPs  2.2. Feedback on medication adherence  Blood pressure monitoring  *It consisted of prompts regarding the patient’s current blood pressure measurements* | Usual cardiovascular management |
| Widmer et al., 2017 | Diet  *Participants used the online and smartphone-based CR program within 1 week following enrolment and entered their diet habits with the help of a study coordinator*  Physical activity  *Participants used the online and smartphone-based CR program within 1 week following enrolment and entered their physical activity with the help of a study coordinator*  Medication adherence  *Participants used the online and smartphone-based CR program within 1 week following enrolment and entered their medication adherence with the help of a study coordinator*  Smoking  *Participants used the online and smartphone-based CR program within 1 week following enrolment and entered their smoking status with the help of a study coordinator*  *Briefly, the DHI encapsulated reporting of dietary and exercise habits throughout CR as well as educational information toward patients' healthy lifestyles* | 3.1. Social support unspecified  2.2. Feedback on behaviour  2.7. Feedback on BP  *Patients who entered values for metrics (such as blood pressure, lipids, glucose, or weight) that were more than 2-fold higher their prior value, or 2 standard deviations above the mean of the normal limits for lab values, were asked to verify the intended entry. If patients confirmed these values, messages to consult their physician appeared; as such changes in weight, blood pressure, or lab values could represent a potential danger to their health*  Blood pressure monitoring  *Participants used the online and smartphone-based CR program within 1 week following enrolment and entered blood pressure with the help of a study coordinator* | Usual care |
